# The Homing Frog: High Homing Performance in a Territorial Dendrobatid Frog *Allobates femoralis* (Dendrobatidae)

**DOI:** 10.1111/eth.12116

**Published:** 2013-07-08

**Authors:** Andrius Pašukonis, Max Ringler, Hanja B Brandl, Rosanna Mangione, Eva Ringler, Walter Hödl, T Tregenza

**Affiliations:** * Department of Cognitive Biology, University of ViennaVienna, Austria; † Department of Integrative Zoology, University of ViennaVienna, Austria; ‡ Department of Tropical Ecology and Animal Biodiversity, University of ViennaVienna, Austria; § Konrad Lorenz Institute of Ethology, Department of Integrative Biology and Evolution, University of Veterinary Medicine ViennaVienna, Austria

## Abstract

Dendrobatidae (dart-poison frogs) exhibit some of the most complex spatial behaviors among amphibians, such as territoriality and tadpole transport from terrestrial clutches to widely distributed deposition sites. In species that exhibit long-term territoriality, high homing performance after tadpole transport can be assumed, but experimental evidence is lacking, and the underlying orientation mechanisms are unknown. We conducted a field translocation experiment to test whether male *Allobates femoralis*, a dendrobatid frog with paternal extra-territorial tadpole transport, are capable of homing after experimental removal, as well as to quantify homing success and speed. Translocated individuals showed a very high homing success for distances up to 200 m and successfully returned from up to 400 m. We discuss the potential orientation mechanisms involved and selective forces that could have shaped this strong homing ability.

## Introduction

Amphibians are among the most sedentary vertebrates with daily movements often reduced to just a few tens of meters (for a review, see Russell et al. 2005; Wells 2007). Despite their lethargic nature, many amphibians occasionally show movements, such as spring migration, over longer distances, ranging a few hundred meters or more. This behavior requires a set of specialized orientation skills. Urodeles, for example, have served as a major model in unraveling vertebrate navigation based on magnetic map sense (Phillips 1996), while field studies on bufonid anurans have revealed multisensory orientation systems that can rely on different sensory modalities depending on cue availability (Ferguson 1971; Sinsch 1987).

Homing performance after experimental translocations from home or breeding sites has been widely used to study animal orientation (Müller & Wehner 1988; Walcott 1996). Translocation experiments not only provide information on a species’ ability and motivation to home back but also can suggest potential orientation mechanisms. In amphibians, translocation experiments indicate the use of spatial maps at least by some species, but the nature of these maps remains unclear (Sinsch 2006). On the one hand, some newt species show remarkable homing from unfamiliar release sites several kilometers away (Twitty et al. 1964), an ability most likely based on magnetic map sense (Phillips et al. 1995). On the other hand, the orientation of other amphibian species is restricted to potentially familiar areas where some sort of landmark learning must occur (Sinsch 1987, 2007).

In anurans, research on homing ability has focused almost exclusively on nocturnal species of the temperate regions, especially bufonids (Bogert 1947; Dole 1972; Sinsch 1987) (but see Gonser & Woolbright 1995; Nowakowski et al. 2012 for work on other groups). Regarding methodology, Wells (2007) pointed out that a control group, quantifying the recapture rate without displacement, is often missing in anuran translocation experiments, which makes it hard to estimate the return success, as a high recapture potential of returning animals is crucial for reliable measurements of return success and speed. However, this criterion is hard to meet for most anuran species in the field without using specialized tracking equipment. Consequently, few studies have reported reliable homing success measures or homing speed as a function of translocation distance (but see Dole 1968, 1972; Matthews 2003). All in all, comparative data of homing performance in anurans are scarce (for an overview, see Wells 2007).

Dendrobatidae (dart-poison frogs) are a group of Neotropical frogs that contrast with temperate region anurans in many aspects of their behavior and ecology. Dendrobatid frogs are diurnal, and they show a prolonged breeding season during which they defend territories. Their life cycle includes terrestrial clutches and parental care such as tadpole transport (reproductive mode #14 *sensu* Duellman & Trueb 1994; Weygoldt 1987). Consequently, dendrobatids exhibit some of the most complex spatial behaviors among amphibians. Despite this fact, orientation mechanisms have not been investigated in dendrobatid frogs. Reliable homing by tadpole-transporting adults is often implicit in studies of species that perform extraterritorial tadpole transport (Roithmair 1994; Ringler et al. 2009), but experimental evidence is lacking.

*Allobates femoralis* (Dendrobatidae) is a dendrobatid leaf litter frog common throughout the Amazon basin and the Guiana shield (Amézquita et al. 2009). Males occupy long-term territories that are vocally advertised and used for oviposition by females (Ringler et al. 2012). Tadpoles are later transported by males to aquatic deposition sites outside their home territory (A, Pašukonis, M. Ringler, H. B. Brandl, R. Mangione, E. Ringler & W. Hödl, pers. obs.; Roithmair 1992; Ringler et al. 2009). We conducted a field translocation experiment to test whether male *A. femoralis* are capable of homing after being displaced as well as to quantify their homing speed and success.

## Materials and Methods

### Study Animals and Area

*Allobates femoralis* is a small (snout-urostyle length approximately 25 mm) territorial dendrobatid frog. At the onset of the rainy season, males establish multipurpose territories, which are vocally advertised and defended for up to several months (Roithmair 1992; Ringler et al. 2009). Playback of an advertisement call of a simulated intruder reliably elicits antiphonal calling or direct phonotactic approach by the resident male (Hödl 1987). Individual frogs can be identified and recognized by their unique ventral coloration patterns (Ursprung et al. 2011b).

**Figure 1:**
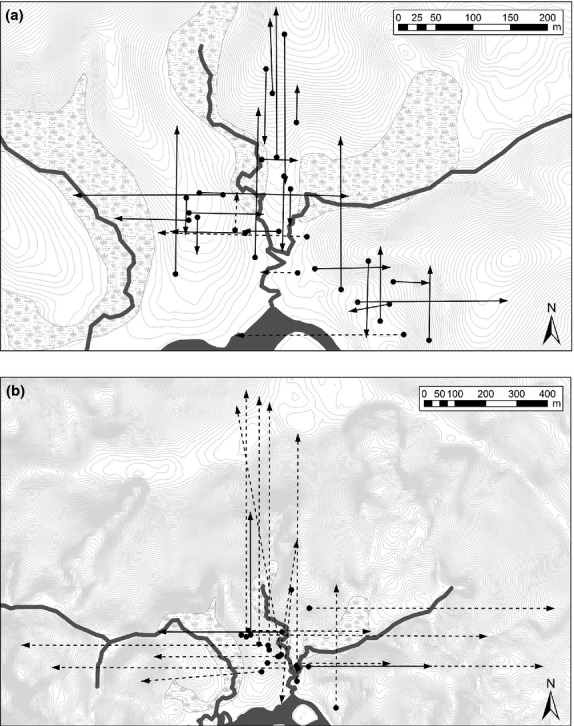
Map of the study area showing all translocations of (a) 50–200 m and (b) 400–800 m. Circles show male capture locations and arrowheads point to the release sites. Solid lines mark successfully retuned and dashed lines non-returned individuals. Contour lines (1 m) are in light gray; creeks and Arataye River in dark gray; palm swamps marked as tussock on the white background.

The study was carried out within one reproductive season of *A. femoralis* between Feb. 25, 2012 and April 4, 2012. Frogs were sampled from a single local population in an area of approximately 15 000 m^2^ near the field camp ‘Saut Pararé’ (4°02′N, 52°41′W, WGS84) in the nature reserve ‘Les Nouragues’, French Guiana. The study area consists mainly of primary lowland rainforest bordering the ‘Arataye’ river to the south. Typically for the rainforest of the Guiana shield, the area has a complex relief (30–140 m asl) composed of small hills and ridges covered in ‘terra firme’ forest and partitioned by numerous small creeks in the lower parts. Flat portions of the creeks form wet areas that are overgrown by açaí palms (*Euterpe* sp.) (Fig. 1). *Allobates femoralis* is only found in the drier ‘terra firme’ forest.

### Translocation Experiment

Translocations took place from Feb. 25, 2012 to Mar. 14, 2012. During this period, 50 territorial males were captured and translocated at equal numbers (10 per translocation distance) to five distances (50, 100, 200, 400, and 800 m) in all cardinal directions (north, east, south, and west). Additionally, 10 control individuals were captured and released without translocation (0 m). All frogs were identified by comparing digital images of their unique ventral coloration patterns.

Calling males were detected and identified as territorial if they showed stereotypical territorial defense behavior (calling and phonotactic approach), which was elicited by broadcasting conspecific advertisement calls, simulating an intruder. Frogs were captured with transparent airtight plastic bags and placed in an opaque container until release. Precise capture positions were recorded with mobile GIS software ArcPad™ 10.0 (ESRI, Redlands, CA, USA) on pocket computers (MobileMapper™ 6, SpectraPrecision, Westminster, CO, USA) using a detailed background map based on a grid of reference points and natural structures. Upon capture, translocation distances and cardinal directions were randomly assigned to the individual frogs. Release points were marked and located using the same detailed background map for distances up to 200 m. More distant release points were located using the GPS of the pocket computers. Translocations southwards were restricted to a single 400-m and to no 800-m release points, due to the proximity of the river on the southern edge of the study area (Fig. 1). All frogs were released within 2 h after their capture. Control frogs were released 10 min–30 min after their capture.

Capture sites of all translocated frogs, as well as of the 10 control individuals, were inspected daily during the peak calling time of *A. femoralis* from 15:00 to 18:00 h (A. Pašukonis, pers. obs.; Kaefer et al. 2013) for at least 14 d or until recapture. Original capture locations of all frogs were first inspected acoustically for calling males in approximately 5 m radius. If no calling individuals were present, a call of a simulated intruder was broadcast from the capture site. All frogs detected during the scanning were captured and identified. Post-14 d, the territories of non-recaptured males were inspected every few days for an additional 6–20 d. Finally, all territories of non-recaptured males were inspected on the 3 and 4 of April.

Five independent variables were measured as potential predictors of return success and speed: translocation distance, direction, water obstacle density, relief complexity, and the frogs’ age. *Translocation distance* was measured as the linear distance from capture to release site. *Translocation direction* corresponded to one of the four cardinal directions (north, east, south, west). *Water obstacle density* was estimated as number of creeks per meter. This measure was taken because *A. femoralis* rather seems to avoid crossing running water (A. Pašukonis, pers. obs.). *Relief complexity* was measured as the ratio between surface (3D) and linear (2D) distances. *Age* was estimated as a binary variable approximating an individual’s age from long-term capture–recapture data of the population (Ursprung et al. 2011a). Frogs were identified by their unique ventral coloration (Ursprung et al. 2011b) and classified as ‘new’ (newly captured frogs in 2012, N = 37) or ‘old’ (recaptures from 2011 and 2010, N = 13). The possibility that some males classified as ‘new’ were older unknown individuals could not be excluded.

### Data Analysis

Return success was statistically analyzed using a multiple logistic regression model with the five independent variables outlined above. Return time was analyzed using a multiple linear regression model with the same independent variables. One frog that was recaptured post-14 d was not considered in the return time analysis because the exact return time was unknown. Surface distances were calculated in ArcGIS™ 9.3 (ESRI), using a topographic map of the area. All statistical analyses were performed with SPSS™ 19.0 (IBM, Armonk, NY, USA).

## Results

Fifty-eight percent of the translocated frogs returned to their home territories within 22 d after the translocation event (Table 1). All control frogs were recaptured inside their home territories within 3 d. While return success was high for translocation distances up to 200 m (87%), it strongly decreased at 400 m (30%), and no frogs returned from 800 m. Logistic regression revealed that translocation distance was the only factor significantly predicting homing success (Table 2).

**Table 1:** Male *Allobates femoralis* homing success after experimental translocation

Translocation distance (m)	N	Homing success (% recaptured)
50	10	80
100	10	100
200	10	80
400	10	30
800	10	0
Total	50	58
Control (0)	10	100

**Table 2:** Output table of the multiple logistic regression model showing a significant correlation between translocation distance and homing success, * p < 0.05

Predictor	Estimate (B)	Standard error	Wald	p
Distance	-0.012	0.004	10.12	0.001*
Direction	-0.004	0.004	0.811	0.368
Relief	-5.9	12.91	0.209	0.648
Water	-98.61	78.21	1.59	0.207
Age	1.82	1.27	2.04	0.153

Model statistics: *R*^2^ = 0.52 (Cox & Snell), 0.7 (Nagelkerke); Model χ^2^(1) = 36.74, p < 0.001.

**Figure 2:**
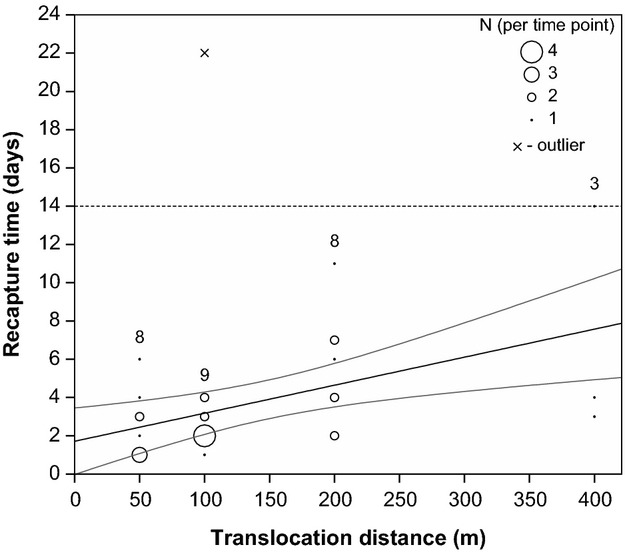
Scatter plot showing the correlation between translocation distance (m) and time until recapture in the home territory (days). Circle size represents the number of individuals, while numbers above each column represent the number of individuals per distance. The regression line with mean confidence intervals is plotted. A single outlier point outside the 14-d daily territory check period is marked by x.

Return time for translocated frogs varied from 1 to 14 d (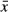 = 3.86, SD = 3), while all control frogs were recaptured within 3 d (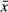 = 1.4, SD = 0.84). Only one frog was recaptured after the daily territory inspection period of 2 wks and was not considered in return time analysis. Minimum return time was 1 d for 50 and 100 m, 2 d for 200 m, and 3 d for 400 m. Maximum return time was 6, 4, 11, and 14 d for 50, 100, 200, and 400 m, respectively. The multiple linear regression model revealed that only translocation distance significantly predicted return time (Table 3; Fig. 2). Translocation direction, water obstacle density, relief complexity, and frogs’ age did not have a significant effect on return time.

**Table 3:** Output table of the multiple linear regression model showing a significant correlation between translocation distance and recapture time, *p < 0.05

Predictor	Estimate (β)	Standard error	*t*	p
Distance	0.527	0.005	3.05	0.006*
Direction	0.108	0.005	0.607	0.550
Relief	−0.006	45.93	−0.032	0.975
Water	0.024	84.54	0.137	0.892
Age	−0.307	1.09	−1.74	0.096

Model statistics: *R*^2^ Linear = 0.372, *F*(5, 27) = 2.61, p = 0.054.

## Discussion

We found that translocated male *A. femoralis* did return to their home territories from up to 400 m within just a few days. Homing performance was high for up to 200 m but steeply decreased for longer distances. Translocation distance was the only factor significantly predicting return time and success. The results of our study not only provide experimental evidence of high homing performance in *A. femoralis,* but they also underline the species’ suitability for studying orientation in dendrobatid frogs. Strong site fidelity, loud advertisement calls, and stereotypic territorial defense behavior make it possible to reliably detect the presence of individuals and thus allow for good estimations of homing success and speed.

It is important to consider the homing distances in relation to the territory size of *A. femoralis* because males spend the vast majority of their time within this area (Ringler et al. 2009). When approximated by a circle, the average area defended by males in our study population is 13.9 m in diameter (cf. Ringler et al. 2011), which is 29 times smaller than the maximum homing distance. Longer homing distances have been reported for some anuran species. Bogert (1947) and Jameson (1957) observed individuals returning from over 1 km in *Anaxyrus* (= *Bufo*) *terrestris* and *Pseudacris* (= *Hyla*) *regilla,* respectively. However, even though the maximal return distance has interesting implications regarding potential orientation mechanisms, a more relevant measure of homing ability is the return success expressed as a percentage of individuals returning from a given distance. To the best of our knowledge, we report the highest homing success of any amphibian for comparable translocation distances.

Failure to return to the home territory can be attributed to orientation failure, loss of motivation, or direct costs such as fatigue and predation. In our opinion, direct costs are unlikely to fully explain the effect of distance on homing success. Predation pressure is relatively low as the majority of the territorial males can be observed conspicuously advertising their presence for several weeks or months from a single location without being predated upon (A. Pašukonis, pers. obs.; Kaefer et al. 2013; Roithmair 1994). Further, male *A. femoralis* routinely transport tadpole loads equivalent to 20% of their body mass to widely dispersed deposition sites (A. Pašukonis, pers. obs.; E. Ringler, A. Pašukonis, W. Hödl & M. Ringler, in prep), a task that is potentially physically more demanding than the return journey alone. It is possible that some frogs returned but were not able to reestablish their territories. However, this effect is probably small as the majority of the territories (16 of 21) of non-returning frogs remained vacant even after 20 d. As the high homing performance across shorter distances indicates a strong general motivation to return, we consider that low homing success for distances above 200 m is best explained by orientation failure.

There are at least five basic orientation mechanisms described in amphibians: path integration, beaconing, piloting, compass orientation, and true navigation (Sinsch 2006). In the present study, all frogs were translocated in opaque airtight bags, and their orientation was changed multiple times during handling and transport. Path integration, which relies on the animal having followed the full outwards path by itself, is disrupted by such experimental translocation. Beaconing is based on a direct sensory contact to the goal, and it could explain homing from shorter but is unlikely for longer distances where direct sensory contact to the home territory is limited. One crucial question in disentangling different orientation mechanisms is whether or not homing is dependent on previous experience with the area. If homing ability relies on experience, a piloting mechanism based on spatial learning of landmarks is implied. If not, this would suggest a true navigation based on map sense, that is, ability to extrapolate long-range directional gradients to position yourself in an unfamiliar area (Phillips 1996).

Our results suggest a homing ability threshold for *A. femoralis* between 200 and 400 m. The ability to home back from such relatively long distances, together with a clear upper limit, is consistent with piloting between landmarks or some sort of local area map. Interestingly, males have been found transporting tadpoles up to 185 m (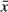 = 38.6, SD = 34.5, N = 129) away from their territory (E. Ringler, A. Pašukonis, W. Hödl & M. Ringler, in prep), which would allow them to explore and learn landmarks in a correspondingly large area. The fact that the distances at which homing success was high are within the range of the longest distances covered by tadpole-transporting adults further supports the idea of experienced based orientation. However, this apparent fit might also suggest that tadpole-transporting distances are limited by orientation ability. Further research should explore the effects of familiarity with an area on the homing ability of dendrobatid frogs in greater detail.

Despite the well-known spatial complexity of dendrobatid behavior, homing has only been investigated in one species (Strawberry poison frog, *Oophaga pumilio*, McVey et al. 1981; Nowakowski et al. 2012). McVey et al. (1981) performed short distance (<20 m) translocations and found a very high homing performance (88% recapture from 12 m, N = 11 and 20 m, N = 6). Contrastingly, Nowakowski et al. (2012) found that only 67% of translocated *O. pumilio* returned from 20 m (N = 30) and 57% from 30 m (N = 30), which is a rather poor performance when compared to *A. femoralis* (87% recapture from 50, 100, and 200 m; N = 30). However, there are several differences in the spatial ecology of *A. femoralis* and *O. pumilio*, most importantly larger territory size and longer tadpole transport distances in the case of *A. femoralis* (Pröhl & Berke 2001; Stynoski 2009; Ringler et al. 2011; E. Ringler, A. Pašukonis, W. Hödl & M. Ringler, in prep.). Further, in *O. pumilio* females perform the tadpole transport and in addition return to the tadpole deposition sites to feed their offspring with unfertilized eggs (Weygoldt 1980; Brust 1993). Consequently, in *O. pumilio* females can be expected to have better orientation ability. Unfortunately, the study of McVey et al. (1981) does not provide a sufficient sample size to draw conclusions in this respect, while the study of Nowakowski et al. (2012) does not specify the sex of translocated individuals.

Attending multiple clutches could have played a major role in selecting for a high homing performance in *A. femoralis* males, which were found to have up to five clutches at the same time (Ursprung et al. 2011a). In such cases, failure to return to the territory after tadpole transport would result in the loss of the other clutches and thus in a severely reduced reproductive output. If extra-territorial tadpole transport and attending multiple clutches were a major selective force in shaping orientation mechanisms, we should find that dendrobatid species lacking these traits would not perform as well in a homing task. The relatively low homing performance of *O. pumilio*, a dendrobatid frog with shorter tadpole transport distances, observed in the study by Nowakowski et al. (2012) is consistent with this hypothesis, but more rigorous comparative work is necessary.
